# A Scoping Review of Fluorescence Imaging: A Promising New Technology for Bacterial Detection in Burn Wounds

**DOI:** 10.1093/jbcr/iraf173

**Published:** 2025-09-13

**Authors:** Steven L A Jeffery, Erik Hanson-Viana

**Affiliations:** Wound Healing Practice Development Unit, Birmingham City University, Birmingham B4 7BD, United Kingdom; Department of Plastic and Reconstructive Surgery, Dr. Rubén Leñero General Hospital, México City 11340, Mexico

**Keywords:** burns, fluorescence imaging, bacterial fluorescence, infection

## Abstract

Burns are complex injuries with devastating long-term impacts. Despite advancements in burn care, infections remain the leading cause of morbidity and mortality. Early and accurate detection of bacterial burden is critical for effective intervention, yet traditional diagnostic methods have limitations. Fluorescence imaging has emerged as an effective tool to enhance bacterial detection and guide infection management in burn wound management. This scoping review summarizes current evidence on fluorescence imaging-guided detection of bacterial loads in burn wounds and explores its potential role across different stages of burn care. A comprehensive literature search was conducted in PubMed using the inclusion and exclusion terms (“fluorescence” OR “autofluorescence”) AND (“burn” OR “burns”) AND “imaging” AND “bacteria” NOT “microscopy.” This search yielded 30 publications, which were further filtered to exclude preclinical studies, review articles, or articles that were not specific to burns. A total of 6 articles investigating the diagnostic accuracy of fluorescence imaging (MolecuLight) in patients with burn injuries were identified. This evidence suggests that fluorescence imaging improves the accuracy of bacterial detection in burns compared to clinical assessment alone, facilitating targeted wound sampling and debridement, enhancing antimicrobial stewardship, and guiding timely interventions. In addition, burn wound surgical planning may be optimized by fluorescence imaging-guided identification of areas requiring excision and grafting. Fluorescence imaging shows promise in enhancing bacterial detection in burn wounds, aiding clinical decision-making and infection management. However, further statistically powered studies are needed to evaluate its impact on patient with burn injury outcomes.

## INTRODUCTION

Burns are a severe and complex form of trauma that have long-term physiological impacts persisting well after the initial injury, imposing significant socioeconomic burdens. Globally, an estimated 8.3 million new cases of burns requiring medical attention occurred in 2019 alone, altogether leading to 111 000 deaths.^1^ In the United States, an estimated $4.1 billion was attributed to burn-related medical costs in 2020, representing an increase of $3.1 billion over a decade and likely an underestimation of the true economic impact.^2^ Though advancements in critical care and therapeutics have improved outcomes, infections remain a serious cause of morbidity and mortality, where approximately 60%-75% of burn mortalities can be attributed to infectious complications.^3-6^

Infections in patients with burn injuries can be devastating as the hallmark loss of a protective skin barrier creates ample opportunity for wound contamination from constant exposure to pathogens. Burn-induced alterations in immune function further predispose patients to infections, resulting in longer hospitalization, which then greatly increases the risk of sepsis and subsequent multiorgan failure.^7^ Early and accurate diagnosis of pathogenic bacteria in and around burn wounds is paramount to prevent further complications and death. Routine surveillance of burn wound infections typically involves evaluating classic signs and symptoms (CSS), followed by confirmation through microbial sampling via surface swabs and less commonly, tissue biopsy. However, there are growing concerns regarding the reliability of these standard practices.^8^^,^^9^

Systemic physiological changes attributed to the burn itself frequently occur in parallel or even without CSS of infection, thus hindering the diagnosis of infection and sepsis in a timely manner.^10^ This issue is especially challenging in patients with extensive burns covering greater than 20% total body surface area (TBSA) who exhibit a more intense systemic inflammatory response, including elevated white blood cell counts, tachycardia, and edema.^10^ These criteria used to screen sepsis in the general population are overwhelmingly present in patients with severe burns, regardless of infection status. Moreover, current guidelines used to diagnose sepsis, such as criteria established by the American Burn Association (ABA)^11^ and the Surviving Sepsis Campaign (Sepsis-3),^12^ do not have strong predictive capabilities compared to prospective clinical diagnoses.^13^ While microbial sampling can provide further information, many limitations exist. Sampling may miss certain areas of bacterial presence, especially if there are bacterial clusters that are not evenly distributed around a large burn area without CSS, making selective sampling difficult, or if they are encased within a biofilm. Sampling larger burns present a particularly challenging issue in determining whether multiple tissue biopsies are indicated, and if so, where the sampling should occur. In addition, swab and tissue biopsy results take several days, by which point the wound environment may have drastically changed, leading to delayed or even inaccurate treatment.^6^ Taken together, there is a clear need for additional tools to supplement the detection and accurate diagnosis of infections in patients with burn injuries.

Recently, point-of-care fluorescence wound imaging technology was developed as a tool to enhance standard-of-care assessments (ie, CSS and microbial sampling) in detecting high bacterial presence in planktonic and biofilm form.^14-16^ This handheld, noninvasive imaging system provides real-time detection of harmful bacteria at levels above 10^4^ colony forming units (CFUs) per gram, loads which are linked to infection and impaired healing.^17-21^ Initially validated in chronic wounds, it has since been shown in numerous studies to enhance the detection of elevated bacterial loads across diverse wound care settings.^14^^,^^22^^,^^23^ This scoping review summarizes the current evidence on fluorescence imaging-guided detection of elevated bacterial loads in burn wounds and explores its potential applications across all stages of burn wound management.

## PATHOPHYSIOLOGY OF BURN WOUNDS AND CURRENT STANDARD OF CARE

A burn wound comprises 3 zones: the central zone of coagulation, where irreversible tissue damage forms an eschar; the intermediate zone of stasis, which is viable with sufficient perfusion and edema control; and the outermost zone of hyperemia, marked by vasodilation and inflammation.^24^ The degree-of-burn severity is based on depth and size (%TBSA), which are the 2 largest outcomes determinants for patients with burn injuries.^25^ Burn depth may be superficial (first-degree), superficial partial or deep partial (second-degree), or full thickness (third-degree); the latter 2 categories represent burns that deeply affect the dermis, require surgery, and have an increased risk of infection.^26^^,^^27^ Importantly, the extent of a burn greatly impacts the pathophysiological response.^28^ Patients with severe burn injuries exhibit a profound inflammatory and hypermetabolic response, often persisting for years after the initial insult, which can impair wound healing and increases the incidence of infections and death.^29^ Therefore, the highest priority for patients with severe burns is to limit wound bacterial colonization to both prevent infectious complications and increase the success of reconstructive procedures.^30^

Thermal injuries disrupt the skin barrier and eliminate resident flora, often leaving burn wounds initially sterile. However, Gram-positive bacteria, particularly *Staphylococcus aureus* (including the highly virulent methicillin-resistant strain, MRSA), typically colonize burn wounds within 5 days, often followed by Gram-negative colonization.^31-33^  *Pseudomonas aeruginosa* is a Gram-negative pathogen that is abundant in burn wounds and poses significant concern due to its propensity to form antimicrobial resistant biofilms.^34^^,^^35^ Of particular concern is the colonization of multiple-drug resistant (MDR) organisms within biofilms, typically produced by MDR strains of *S aureus*, MRSA, and *P aeruginosa.*^36^ Biofilm-associated sepsis is well-documented, therefore, it is crucial to detect and mitigate high bacterial loads in burn wounds during early, preinfection phases (bacterial levels are below 10^5^ CFU/g).^11^^,^^37^

Incorporating early excision and skin grafting into standard burn wound care practices has greatly reduced the incidence of infection, sepsis, and mortality.^38^ Optimal outcomes are achieved when excision is performed as soon as possible, ideally within 48 h postburn, as early intervention mitigates the risk of hypermetabolic and hyperinflammatory responses while reducing infection risk.^39^^,^^40^ Following complete debridement of necrotic tissues from the wound bed, preparation of the wound bed must be ensured with temporary allografts, xenografts, or skin substitutes, for later application of skin autografts.^40^ Autologous split-thickness skin grafts (STSG) are considered the gold-standard for burn management, but temporary skin substitutes are recommended if donor sites are limited or if bone and tendons are exposed. This is because bioengineered skin substitutes can stimulate the formation of well-perfused granulation tissue over exposed structures, enhancing success rates of subsequent grafting procedures.^41^ Several artificial skin substitutes composed of biological, synthetic, or biosynthetic materials have been developed with some success, though many are costly or lack sufficient data.^27^

Despite advances in skin grafting, take rates have not significantly improved since the 1940s, largely due to infection.^42^^,^^43^ The mere presence of high bacterial loads alone is a significant predictor of skin graft failure and is associated with increased morbidity and mortality.^44^^,^^45^ Therefore, early and accurate detection of wound contamination is essential to minimize costs, save time, and reduce the risk of poor outcomes. Standard practices for diagnosing burn wound infections are based on subjective (CSS) and objective (microbial sampling) measures. As outlined by the ABA, rapid changes in the burn wound appearance, such as erythema, color changes, odor, eschar separation, and graft loss, and changes in the patient’s condition, such as pain, fever, tachycardia, and thrombocytopenia, trigger a clinical concern for infection.^11^ In addition, quantitative microbial sampling via tissue biopsy or superficial wound swabs must detect greater than 10^5^ CFU/g bacteria to confirm the presence of infection. While tissue biopsies accompanied by histological examinations are considered “gold-standard” due to their accuracy,^46^ wound swabs are preferred for burn wounds given their low cost, noninvasiveness, and convenience, particularly for larger burns or areas with thin skin.^6^

Despite existing guidelines, diagnosing burn wound infection remains challenging for several reasons. First, subjective clinical findings are often unreliable as all patients with major burn injuries exhibit persistent systemic inflammatory responses that are also associated with infection and sepsis in the general population.^11^^,^^40^ Moreover, changes in wound appearance, such as color or erythema, may be less obvious in patients with darker skin.^47^ Second, the use of microbial sampling, while objective in nature, may not be ideal for patients with burn injuries due to limitations in timing and accuracy. Regardless of tissue biopsy or wound swab, results are not immediate and may take days to process, at which point, the microbiome of the wound may be drastically altered.^9^ Superficial wound swabs are also limited in their capability to detect biofilms.^48^ Lastly, especially when managing larger burns with suspected infection, only a limited number of sites may be sampled, and these are usually selected subjectively. To circumvent these issues, fluorescence-guided sampling has been implemented to enhance current practices and subsequently improve patients with burn injuries outcomes.

## BACTERIAL FLUORESCENCE IMAGING IN BURNS

**Figure 1:**
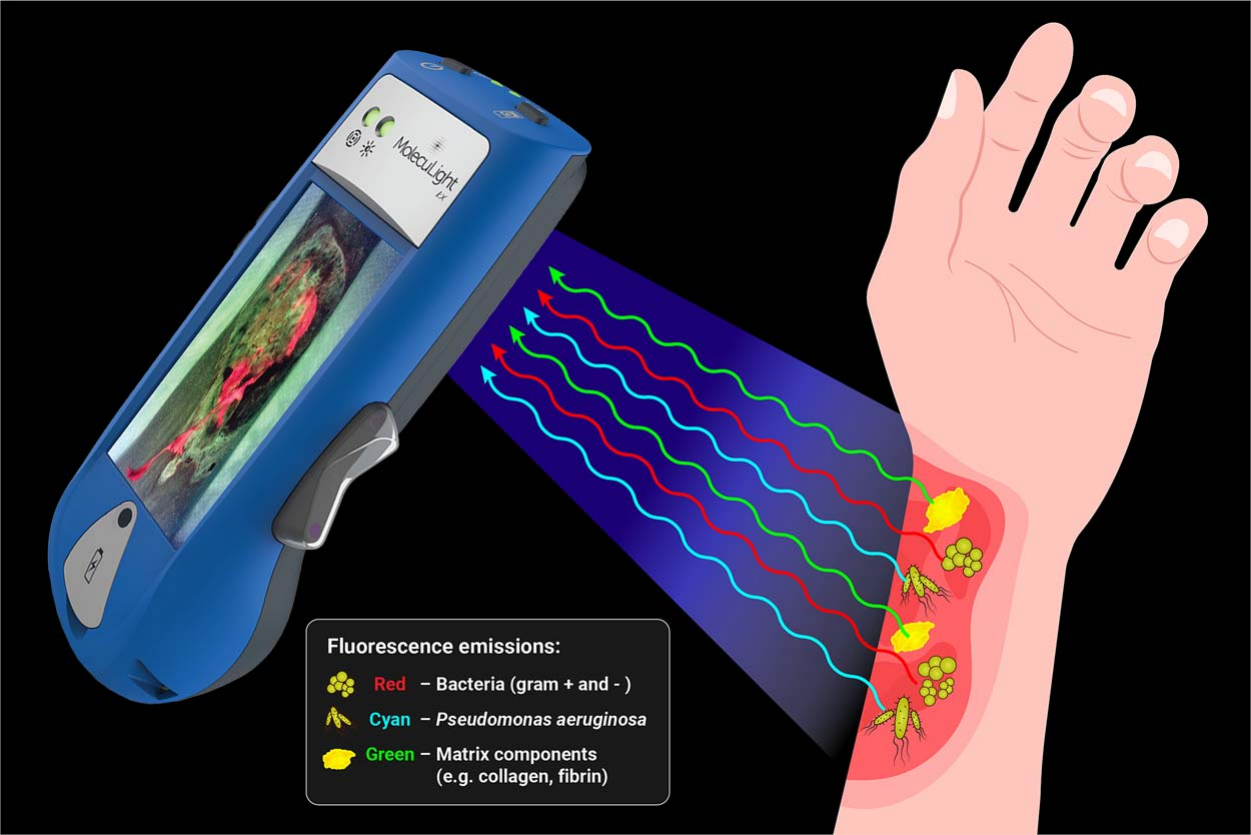
The Fluorescence Imaging Device Illuminates a Burn Wound With a Safe Violet Light (405 nm) Resulting in the Excitation of Elevated Bacterial Loads (>10^4^ CFU/g) and Tissue Components. Red Fluorescence Signals Indicate the Presence of Gram-Positive or Gram-Negative Bacteria, While Cyan Fluorescence Signals Indicate *P. aeruginosa*. Normal Tissue Matrix Components (eg, Collagen, Fibrin) Emit a Green Fluorescence Signal. These Signals Are Captured by the Device and Interpreted by a Clinician to Identify the Presence and Location of Bacteria and/or Biofilm for Targeted Treatment

Fluorescence imaging tools have emerged as a method for bacterial visualization in various wound care settings. The fluorescence imaging procedure is completely noninvasive, yet can detect planktonic and biofilm-encased bacteria at a depth of up to 1.5 mm into the skin.^14^^,^^49^^,^^50^ These devices emit a safe violet light (405 nm) onto wound tissues from a short distance, stimulating the emission of a wide spectrum of fluorescence signals from endogenous bacterial components (namely, pyoverdines and porphyrins) and tissue matrix components (eg, collagen, elastin) (Figure 1). The signals are passed through an optical filter built into the device, generating real-time fluorescence visualization on the device screen, which can be saved as images or videos in the patient record. These images contain red, cyan, or green fluorescence colors that require interpretation by a clinician. Red and cyan colors indicate the presence of a moderate-to-heavy bacterial load (>10^4^ CFU/g), where red fluorescence is produced by the accumulation of a range of porphyrin-producing bacterial species (eg, *S aureus*, *Escherichia coli*, and *Klebsiella pneumoniae*) and cyan fluorescence is specific to pyoverdine-producing *P aeruginosa.*^16^ These fluorescent patterns differentiate bacterial species from native tissue structures, which will fluoresce green, corresponding to normal skin components.

Real-time fluorescence imaging technology was developed to address the various unmet needs of standard-of-care procedures. Its applications and benefits are well described for the chronic wound population,^15^^,^^19^^,^^51^^,^^52^ where fluorescence imaging significantly enhances the sensitivity of bacterial detection by approximately 4-fold.^14^ This immediate bacterial visualization aids clinicians in quickly implementing targeted treatment plans, which improves clinical outcomes and reduce treatment costs.^14^ Indeed, real-world and prospective studies report faster healing with 23%-104% increases in chronic wound healing rates, occurring alongside significant reductions in antibiotic use, complications risk, and antimicrobial dressing expenditure.^52-54^ Clinicians report using the incremental information from fluorescence imaging to guide important decisions concerning pre-, intra- and postoperative care for both chronic and acute wounds.^14^^,^^55^^,^^56^ Wound hygiene and debridement, both critical components of wound bed preparation, can be guided by fluorescence imaging for increased efficacy. This is well-described in a study by Moelleken et al. (2024), which reported that significantly more bacteria were removed when using fluorescence imaging to guide debridement compared with unassisted methods.^57^ Best practice consensus guidelines developed by a multidisciplinary panel of wound experts recommend that fluorescence-assisted debridement be used to remove all bacterial-laden tissues before proceeding with advanced therapies such as skin substitutes and negative pressure therapy.^58^ These guidelines underscore the broad utility of and substantial evidence for fluorescence imaging in improving the care of complex, nonhealing wounds.

## LITERATURE SEARCH METHODOLOGY AND RESULTS

Growing evidence suggests that fluorescence imaging may also confer similar benefits of improved bacterial detection and more objective management for burn injuries. To amass and characterize this evidence, a comprehensive search for peer-reviewed articles focusing on the use of fluorescence imaging in burn care was conducted in PubMed. Reports were identified using inclusion and exclusion terms (“fluorescence” OR “autofluorescence”) AND (“burn” OR “burns”) AND “imaging” AND “bacteria” NOT “microscopy.” This search yielded 30 publications, which were further filtered to exclude preclinical studies (*n* = 20), review articles (*n* = 2), or articles that did not report diagnostic accuracy measures specific to burns (*n* = 2). A total of 6 articles investigating the diagnostic accuracy of fluorescence imaging in patients with burn injuries were identified and reviewed in full.^59-64^

Table 1 summarizes the design, objectives, results, and conclusions of the 6 articles included in this review. These were all single-center retrospective or prospective studies that employed the MolecuLight fluorescence imaging system to evaluate burn wounds. Our search did not identify any additional fluorescence imaging devices used in the evaluation of bacteria in burn wounds. The populations represented included adult and pediatric patients with burn injuries, and the study sample sizes ranged from 3 to 63 patients (14-77 wounds imaged). Most studies were aimed at determining the diagnostic accuracy measures of fluorescence imaging for detecting high bacterial loads relative to microbial swabbing.^59-61^^,^^63^^,^^64^ However, Hanson-Viana et al. (2024) report diagnostic accuracy measures of fluorescence in terms of predicting STSG loss/take.^62^

**Table 1:** Summary of Studies Using Fluorescence Imaging to Detect High Bacterial Loads in Burn Wounds

**Author, year**	**Study design**	**Study objectives**	**# Patients/Wounds assessed**	**Sensitivity**	**Specificity**	**PPV**	**NPV**	**Accuracy**	**Conclusions**
Hanson-Viana et al., 2024	Single-center prospective observational study	FL of wounds before graft placement with surgical team blinded; assess graft loss/take over FL-positive (+) and -negative areas (−); true positive was determined as failed grafts with FL+ and false negatives referred to failed grafts that were FL−.	38 patients. 38 wounds imaged.	86%	98%	72%	99%	94%	FL predicted skin graft outcomes with high accuracy, particularly graft loss. FL is recommended as a highly reliable decision-making tool.
Turner et al., 2024	Single-center retrospective chart review	Subanalysis of 178 pediatric patients; compare FL + CSS with microbial swabs (Levine or targeted to FL); assess diagnostic accuracy of burn-originated infection detection via FL.	Subanalysis: 63 patients. 77 wounds imaged.	78%	71%	26%	96%	71%	FL correlated well with swabs and improved the detection of infection beyond what would be detected by CSS alone. Considerable benefits to adding FL to standard practices.
Farhan and Jeffery, 2020	Single-center prospective observational study	Evaluate diagnostic accuracy and practicality of FL in pediatric burn wounds positive for CSS of infection; compare FL findings with swab results (Levine or targeted to FL).	10 pediatric patients. 16 wounds imaged.	100%^a^	73%^a^	63%^a^	100%^a^	81%^a^	FL detected clinically significant bacterial loads with high sensitivity. High compliance rate among patients and clinicians. Recommended to use as an aid for current practices.
Pijpe et al., 2019	Single-center prospective observational study	Evaluate diagnostic accuracy of FL in burn wounds; compare swab results (Levine, FL-guided, and FL-combined) with the presence of red and/or cyan FL areas.	14 patients. 20 wounds imaged.	Red and/or cyan: 78% Cyan: 100%	Red and/or cyan: 64% Cyan: 70%	Red and/or cyan: 64% Cyan: 44%	Red and/or cyan: 78% Cyan: 100%	Red and/or cyan: 70%^a^ Cyan: 75%^a^	FL had moderate diagnostic accuracy with reliability equal to standard swabs. Especially useful in ruling out high presence of *P aeruginosa*.
Alawi et al., 2018	Single-center prospective observational study	Evaluate diagnostic accuracy of FL in detecting burn infections following STSG; compare swab results (targeted to FL) with FL findings.	3 patients. 14 wounds imaged.	87%	88%	82%	90%	87%^a^	FL useful as an addition to current practices to assess bacterial load in skin grafts.
Blumenthal and Jeffery, 2018	Single-center prospective observational pilot study	Use FL to guide swabbing in burn wounds. Correlate findings of bacterial presence via FL and swab results.	20 patients. 20 wounds imaged.	81%^a^	75%^a^	93%^a^	50%^a^	80%^a^	The efficacy of FL is evident due to the microbiology results correlating to the images.

Abbreviations: CSS: classic signs and symptoms; FL: fluorescence imaging; STSG: split-thickness skin grafts.

^a^Calculated manually based on reported positive microbiology results as standard of care.

### Diagnostic accuracy of fluorescence imaging in burns

The diagnostic accuracy reported for fluorescence imaging of burn wounds ranged from 70% to 87% relative to microbial swabbing (ie, targeted to fluorescence or conventional Levine technique).^59-61^^,^^63^^,^^64^ Sensitivities ranged from 78% to 100% and specificities ranged from 64% to 88%. Turner et al. (2024) conducted the largest study (retrospective review of 77 pediatric burn wounds), reporting that FL + CSS improved sensitivity by 39% compared to CSS alone (56% vs 78%).^63^ However, this resulted in a 19% reduction in specificity (88% vs 71%), which the authors speculate could be due to the detection of fluorescence-positive areas that did not correspond to microbial findings. This raises the question of whether fluorescence imaging can identify clinically significant bacterial loads that may be missed by conventional swabbing. For example, Blumenthal and Jeffrey (2018) previously described a case example whereby a patient demonstrated CSS (thick exudate and slough) in the center of the burn wound.^60^ However, the presence of red fluorescence and positive microbial swabbing identified elevated bacteria on the surrounding tissue, which would have been overlooked by conventional methods alone. A similar case report of a pediatric burn wound described by Farhan and Jeffrey (2020; Patient #3) further supports this notion.^64^ Collectively, these studies conclude that fluorescence imaging has considerable diagnostic accuracy, sensitivity, and specificity for detecting bacterial loads in burns, and that fluorescence has potential to enhance bacterial detection and microbial swabbing techniques above the current standard of care.

Positive and negative predictive values (PPV and NPV, respectively) are also useful in assessing diagnostic reliability. Among the articles reviewed, PPV for fluorescence imaging in burns ranged from 26% to 93% (Table 1).^59-64^ This wide range could be related to prevalence of fluorescence in burns; the vast majority of burns across all studies were fluorescent-negative (12%-70% fluorescent-positive), meaning that lower PPV values may have been due to limited data points. Despite variability in PPV, fluorescence imaging has demonstrated consistently high NPV (78%-100%) across burn-related publications, barring an early pilot study (50%).^60^ In that instance, the authors note that while the microbial swab results were negative, the device showed positive fluorescence signals within deeper tissue folds that demonstrated CSS, resulting in a low NPV. False negatives were uncommon across all studies, suggesting that if the device does not detect red or cyan fluorescence, it is likely that a clinically significant bacterial load is absent. Overall, these additional measures of diagnostic performance further support the authors’ conclusions that fluorescence imaging is generally reliable for the detection of clinically relevant bacteria in burn wounds. Accordingly, the majority of studies highly recommended the implementation of fluorescence imaging devices to aid in the detection of elevated bacterial burdens together with CSS and microbial sampling in adult and pediatric burn wounds.

### Guidance and predictive capability for skin grafting

Split-thickness skin grafts are frequently used to restore the skin barrier and mitigate the risk of serious complications in patients with severe burn injuries. Despite advancements over the past 7 decades, graft failure rates remain significant, ranging from 5% to 18%, largely due to bacterial contamination.^43^^,^^65^^,^^66^ While just 2 studies describe the use of fluorescence imaging-guided STSG in burn wounds, these early results demonstrate promising outcomes. A pilot study by Alawi et al. (2019) examined 14 burn wounds treated with STSG in 3 patients and found strong correspondence of fluorescence imaging with microbial sampling, resulting in a considerable accuracy of 87%.^59^ More recently, Hanson-Viana et al. (2024) evaluated the predictive capability of fluorescence imaging in assessing skin graft integration by detecting elevated bacterial presence prior to STSG application in 38 adult patients with burn injuries.^62^ Notably, fluorescence imaging detected bacteria (>10^4^ CFU/g) in all patients with partial graft loss that conventional microbial swabbing missed, with graft success rates per cm^2^ of 99.2% in fluorescent-negative areas compared to 27.9% in fluorescent-positive areas. In addition, the fluorescence imaging device predicted graft loss with a sensitivity and specificity of 86% and 98%, respectively, and achieved 94% accuracy in predicting graft loss overall, independent of burn size. These findings suggest that fluorescence imaging is a highly accurate tool in predicting skin graft outcomes, particularly in fluorescent-negative areas, potentially reducing complications and improving functional and cosmetic outcomes for patients with burn injuries.

### Impact on patient compliance and burn wound management

Although most of the articles reviewed focused on assessing the diagnostic performance of fluorescence imaging in burns, the impact of this technology on patient compliance and overall burn wound management is also described. Farhan and Jeffrey (2019) conducted a clinician questionnaire regarding fluorescence imaging of pediatric populations.^64^ Patient compliance was reported at 100%, with 89% of pediatric patients with burn injuries experiencing little to no fear or discomfort from the darkened environment during fluorescence imaging.^64^ Nearly all respondents (93%) found the device practical for use in pediatric burn wound care, underscoring the feasibility of fluorescence imaging in this population.^64^ Notably, neither patient engagement nor compliance was addressed in the remaining 5 studies.

When considering the impact of fluorescence imaging on overall burn wound management, some studies describe its utility in guiding wound bed preparation and sampling, with positive implications for antimicrobial stewardship. Turner and colleagues observed that fluorescence imaging led to an increase in wound cleansing procedures, with 31% of burn wounds undergoing a second cleansing following the detection of fluorescence-positive areas.^63^ Blumenthal and Jeffery (2020) report that post-debridement fluorescence imaging was useful in assessing treatment efficacy, noting one burn where bacterial fluorescence was evidenced on the surrounding tissue despite CSS pointing to the wound interior.^60^ Similarly, other studies report that fluorescence prompted swabbing of CSS-negative burns.^60^^,^^63^^,^^64^ For example, positive fluorescence prompted Farhan and Jeffery (2020) to sample a CSS-negative burn, ultimately revealing a covert *S aureus* infection.^64^ Blumenthal and Jeffery (2018) report the same phenomenon, leading the authors to recommend fluorescence-targeted sampling for more accurate and proactive infection detection.^60^ In addition, they suggest that a targeted, fluorescence-informed approach to burn hygiene could prevent infections, ultimately reducing the need for systemic antibiotics and preventing antibiotic resistance.^60^ Hanson-Viana et al. (2024) also strongly recommended fluorescence-guided sampling in the context of targeting and prioritizing skin grafting sites.^62^ Collectively, these studies attribute improved burn wound hygiene and sampling, and ultimately more effective infection management, to fluorescence imaging.

## LIMITATIONS AND FUTURE DIRECTIONS

There are some important limitations to consider. First, the current studies are primarily observational with relatively small sample sizes. Larger studies would improve the generalizability and validity of the diagnostic performance metrics for fluorescence imaging in burns, and additional interventional studies would be useful to study how improved bacterial detection could impact patient outcomes. Second, the swabbing procedures varied between the studies reviewed; some employed the Levine technique (industry standard) while others swabbed areas of positive or negative fluorescence. There is a greater chance of a false negative swab (ie, missing areas of fluorescence) when using the Levine technique, which may have impacted the diagnostic performance measures reported. Furthermore, Pijpe et al. (2019) set the threshold for a positive microbiological sample at 10^2^ CFU/g; this is below the threshold of detection using fluorescence imaging (10^4^ CFU/g) and may therefore have inflated the number of false negatives.^61^ Moreover, 10^2^ CFU/g represents a low level of bacteria observed during early colonization and the presence of bacteria alone is insufficient to cause infection.^11^ To overcome these issues, we recommend that future diagnostic studies sample fluorescence-positive regions exclusively and set the threshold for positive microbiology at 10^4^ CFU/g to yield the most accurate results. This will also improve the homogeneity of the literature base for future reviews. Finally, the studies reviewed herein largely focused on the reliability of fluorescence imaging for bacterial detection in burns. Future research should focus on how integrating this technology into clinical decision-making could lead to improved burn wound management. Future research should also focus on patient engagement and treatment compliance, as these patient-centric factors have been linked to wound-healing success^67^ and are largely absent in the evidence base reviewed.

## CONCLUSION

Burns are complex injuries that significantly increase patients’ vulnerability to infections. Although burn care has advanced over the past few decades, infections remain the leading cause of morbidity and mortality. There is an increased demand for novel technologies that enhance current standard-of-care methods in identifying the presence and location of pathogenic bacteria. Fluorescence imaging has emerged as an effective tool for assessing bacterial bioburden in burn wounds. In this scoping review, we have highlighted the current data assessing the impact of fluorescence imaging on diagnostic accuracy, predictive capabilities in graft outcomes, and patient compliance in burn wound management. These data suggest that fluorescence imaging is a clinically valuable and highly accurate tool that has a positive impact on clinical decision-making at every stage of burn wound care. While further robust studies focusing on patient outcomes are necessary, the current data regarding the association between fluorescence imaging-guided bacterial detection and burn wound management are promising.

## Notes

Author contributions: Steven L. A. Jeffery (Conceptualization, Data curation, Formal analysis, Investigation, Visualization, Writing—original draft, Writing—review & editing [equal]), Erik Hanson-Viana (Conceptualization, Data curation, Formal analysis, Investigation, Visualization, Writing—original draft, Writing—review & editing [equal])

Funding: *This work received no external funding.*
